# Innovative Operations Measures and Nutritional Support for Mass Endurance Events

**DOI:** 10.1007/s40279-015-0396-6

**Published:** 2015-11-09

**Authors:** George T. Chiampas, Anita V. Goyal

**Affiliations:** Departments of Orthopaedic Surgery and Emergency Medicine, Feinberg School of Medicine, Northwestern University and Northwestern Medicine, 211 East Ontario Street, Chicago, IL 60611 USA; Bank of America Chicago Marathon and Shamrock Shuffle, Chicago, USA; US Soccer Federation, Chicago, USA; Saint James School of Medicine, The Quarter, Anguilla

## Abstract

Endurance and sporting events have increased in popularity and participation in recent years worldwide, and with this comes the need for medical directors to apply innovative operational strategies and nutritional support to meet such demands. Mass endurance events include sports such as cycling and running half, full and ultra-marathons with over 1000 participants. Athletes, trainers and health care providers can all agree that both participant outcomes and safety are of the utmost importance for any race or sporting event. While demand has increased, there is relatively less published guidance in this area of sports medicine. This review addresses public safety, operational systems, nutritional support and provision of medical care at endurance events. Significant medical conditions in endurance sports include heat illness, hyponatraemia and cardiac incidents. These conditions can differ from those typically encountered by clinicians or in the setting of low-endurance sports, and best practices in their management are discussed. Hydration and nutrition are critical in preventing these and other race-related morbidities, as they can impact both performance and medical outcomes on race day. Finally, the command and communication structures of an organized endurance event are vital to its safety and success, and such strategies and concepts are reviewed for implementation. The nature of endurance events increasingly relies on medical leaders to balance safety and prevention of morbidity while trying to help optimize athlete performance.

## Introduction

With the increased global popularity of endurance events such as cycling and running half, full and ultra-marathons, meeting the medical demands for the unique conditions seen in these events has become a priority in the realm of sports and mass medical management. Race and medical directors, as well as cities and municipalities hosting these events, have the dual roles of providing medical care to the participants while maintaining public safety. Increasing numbers of participants, in some instances up to 45,000, are utilizing the streets and waterways of small and large cities and can tax even the most advanced health care system. Preparing for the expected, as well as the unexpected—as in the circumstances of the Boston Marathon in 2013—should be part of the operations plan for all events. As a planner, preparing for, managing and responding to the unique medical conditions associated with these mass sporting events is critical to participant outcomes and mitigation of public safety incidents. The conditions to address include collapse of the athlete, heat illness and exercise-associated hyponatraemia (EAH), as well as cardiac incidents, all requiring attention to strategic protocols and athlete fuelling for positive outcomes. Finally, instituting innovative medical processes via a command centre, with protocols, resources and tools for communication, and tracking capabilities that are scalable and specific to the event are critical for overall safety.

When assessing a mass sporting event, defined as an event involving over 1000 participants, certain circumstances should be considered. These include the type of endurance event, participant make-up, time of the year and time of the day. Using historical forecasts is useful to gain a clear understanding that in some circumstances, environmental factors will impact medical encounters upwards from 2 to 10 % of participants based on conditions [1]. Start- and finish-line areas with capabilities to provide on-site care across the course, with a focus on the finish-line area and access to local hospitals, should be determined. The overall scope of care should be discussed with planners in advance, and advanced life-support capabilities should be put in place for initiation, management and triage of medical conditions as needed. The 2013 Pittsburgh ‘Rock ‘n’ Roll’ Half-Marathon was cancelled when the city withdrew the permit for an August event, citing public safety concerns based on potential environmental issues [2]. With increasing popularity and increasing participant numbers, the event should not overwhelm the emergency management and public safety system [3]. On-site medical care, with providers experienced in managing acute endurance-related conditions, can mitigate these and support mass sporting events.

**Fig. 1:**
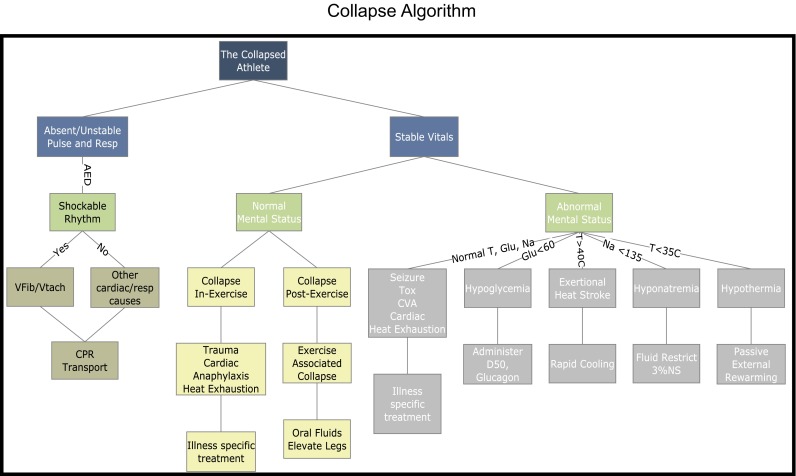
Collapse algorithm. The chart depicts the approach to medical management and decision-making around the collapsed athlete during an endurance event. *AED* automated external defibrillator, *CPR* cardiopulmonary resuscitation, *CVA* cerebrovascular accident, *D50* dextrose 50, *Glu* glucose (mg/100 mL), *Na* sodium (mmol/L), *NS* normal saline, *Resp* respiration, *T* temperature (°C), *Tox* toxicology, *Vfib/Vtach* ventricular fibrillation/ventricular tachycardia. Reproduced from Malik et al. [4], with permission

Medical conditions associated with endurance events may be of the ordinary variety seen in clinical practice; however, there are several specific to endurance medicine that can be potentially life threatening. While upwards of 60 % of conditions seen in endurance events are musculoskeletal in origin, the most concerning and life-threatening conditions are seen with collapse [1]. Rapid identification and appropriate response to various endurance exercise-associated collapses (EACs) is critical for positive outcomes. Most modern-day endurance events are staffed by medical volunteers and/or emergency medical service (EMS) providers with a broad scope of clinical practice. Conditions associated with endurance events are not routinely seen or managed in clinical practice. Therefore, advance implementation of an educational tool for medical personnel, highlighting these conditions, creates consistent and directed care. With recent advances in technology, the options for mass medical education are vast. One programme utilized by the Bank of America Chicago Marathon is a Web-based PowerPoint interactive tool, which is sent to all registered medical personnel 1–2 weeks prior to the event. The tool reviews the Collapse Algorithm (Fig. 1), highlighting the potential extreme issues, with the option to add questions to assess competency [4]. Additional tools utilized are webinars and videos, which can be accessed by or distributed to medical providers with content on best-practice management of endurance-associated conditions on race day.

EAC is the leading injury linked to these events and not commonly seen in the general population. However, it is seen in over 50 % of medical encounters in marathons [1]. Effective regulation of pace enables the majority of runners to complete competitive endurance events without mishap [5]. However some runners do experience EAC associated with postural hypotension, which, in rare cases, results from life-threatening conditions such as cardiac disorders, heat stroke and hyponatraemia. EAC is a relatively benign condition, where venous return is hindered upon sudden cessation of participation. This leads to a plethora of symptoms and ultimate collapse of the athlete. The unique component of EAC is that mental status remains preserved during this condition and, with simple elevation of the athlete’s legs, equilibration of vascular volume occurs over a short period of time, and symptoms abate. During these mass endurance events, medical providers will encounter athletes presenting in the ‘Foster collapse positions’ and must rapidly discern benign versus life-threatening collapse [1, 5]. Additional information regarding the treatment of participants in ultra-endurance events in remote environments is available in a recent review [6].

## Heat Illness

Environmental conditions play a critical part in injuries associated with endurance events, with heat-associated injuries being most serious. During endurance events, participants will experience the spectrum of heat-related issues, with heat stroke being most severe and having the greatest morbidity. Incidence and severity will directly correlate with environmental factors on race day; however, these issues will still be seen on cooler days or shorter courses. During the Tel Aviv Half-Marathon in March 2013, one runner died and 30 were injured because of heat issues [7]. For these reasons, having specific protocols with resources to rapidly cool runners in multiple ways is imperative.

Athletes with symptoms of heat illness will present with similar symptoms of fatigue, nausea and headache, yet with preserved mental status. They need to be removed from the direct environmental conditions and have passive cooling measures started. Athletes with heat stroke are identified by altered mental status, nausea, headache, vomiting, seizures and/or collapse, and require emergency cooling. Having on-site rectal temperature monitoring capabilities at the finish-line medical facility is critical in managing these conditions. Immediate identification and provision of rapid cooling measures with ice submersion is best practice for resolution of symptoms [8]. Utilization of a six-person team for safe submersion is critical for patient safety and airway management during the cooling process, as the athlete may be combative or somnolent. Several methods are currently used to assist in athlete ice submersion. Continuous rectal temperature monitoring is needed, and there are several devices that are being utilized across several mass endurance venues. The athlete should be removed from the ice submersion when their body core temperature decreases to 101.4 °F (38.6 °C), to prevent hypothermia. Having on-course submersion tubs and access to large volumes of water may be cumbersome. Various other techniques are good alternatives in these circumstances. For instance, kiddie pools can be used for immersion, or methods of applying ice massage and/or alternating wet towels to the groin and axilla are good options to cool athletes experiencing heat-related issues. Water-ice therapy is an alternative method to cold-water immersion. With this method, patients are treated while lying supine on a porous stretcher resting over a tub filled with cold water. Medical personnel then douse the patient with water while others massage large muscle groups with ice bags. This method has produced cooling rates that were 70 % as effective as those achieved with immersion, with positive outcomes [9]. Additionally, preventative measures can similarly be incorporated. Implementing communications to participants regarding course conditions forecasts based on weather, as well as acclimation factors associated with participant demographics, are good tools for prevention. Educating runners about the location and use of on-course resources such as towels, sponges or showers, together with the implications of adjustments of pace, should be incorporated for heat-triggered protocols. Finally, ensuring that EMS providers and local receiving hospitals have similar resources in place ready to be implemented or continued on transported athletes are vital for positive outcomes.

## Exercise-Associated Hyponatraemia

A life-threatening cause of collapse during endurance exercise is EAH. In recent years, it has been responsible for six deaths in the USA and UK [10]. EAH is defined as a serum sodium ([Na^+^]) level of <135 mmol/L during or up to 24 h after prolonged physical activity. This condition, however, is further categorized on the basis of acute symptoms as symptomatic or asymptomatic hyponatraemia, as the absolute value of [Na^+^] is not a reliable predictor of clinical severity [10]. Initial symptoms may be subtle, such as bloating, nausea and/or vomiting or headache. As symptoms worsen, athletes may present with hyponatraemic encephalopathy and a spectrum of altered mental status, with potential collapse, seizure, coma and death due to acute cerebral oedema. Studies of marathon runners in Europe and the USA have shown the incidence to be between 3 and 22 % [11]. EAH is understood to be a dilutional hyponatraemia caused by an increase in total body water relative to the amount of exchangeable sodium stores [11]. Risk factors in athletes have been identified as excessive fluid consumption in excess of total body fluid losses, altered renal function, longer race times (typically greater than 4 h), female sex and a low body mass index [12]. Excessive sodium loss may have an additional effect on EAH; however, this has not been identified as a primary factor.

For a race organizer or medical provider, this is a challenging problem. A recent study reported that 64 % of marathon runners are not concerned about the condition of hyponatraemia, while 68 % are not weighing themselves, and over 80 % have no other method of individual hydration assessment [13]. Therefore, several methods should be in place for an event if the risk of EAH exists. First, education of the athletes on potential individual risks based on their profile and abilities should be considered. There is also the need to address human behaviours and educate athletes on preventative measures they can take to minimize issues with hydration during events. Having a hydration plan in place during their training is critical for good outcomes. Measuring body weight before and after a long or simulated event during training is one preventative measure [14]. Athletes should not gain any weight and ideally should not lose more than 2–3 % of their pre-training weight, though EAH has been seen in endurance athletes who have lost weight [15, 16]. During moderate to strenuous training days in various environmental conditions, gauging the volume and types of fluids individually needed is essential for implementation of best and safe performance outcomes.

Medical providers for events should have a good awareness of presenting symptoms of symptomatic hyponatraemia and those at highest risk of the condition. It is critical to rapidly identify athletes with EAH and either transfer them to a medical facility with fluid restrictions en route or have on-site point-of-care testing. Point-of-care testing offers the ability to rapidly measure [Na^+^] on-site. Athletes with mild symptoms and no confusion should be monitored and provided with oral hypertonic solutions. In one study, 16 runners with EAH who were able to drink a concentrated oral hypertonic solution recovered within 30 min [12]. In more severe cases of EAH, with presenting symptoms of confusion, seizures or coma, current consensus guidelines recommend up to three 100 mL boluses of 3 % sodium chloride (NaCl) solution spaced at 10-min intervals to correct symptoms [17]. At endurance events with laboratory capabilities, having hypertonic saline available to initiate care while preparing for hospital transfer may mitigate morbidity. Additional measures to have in place include advance education of EMS providers on the risk of EAH and the importance of restricting intravenous hydration in athletes experiencing EAH symptoms. Also, informing local receiving hospitals of the potential presentation of EAH and the need for rapid management may lead to improved outcomes, as these conditions are rarely seen in the general population.

## Nutrition

Another important component of operations for mass endurance events is nutrition. Nutritional support could potentially serve as a preventative measure, reducing morbidity on race day. Glycogen and glucose are the fundamental energy sources for muscle during races [18]. During long periods of exertion, these are depleted, warranting extra attention to maintain optimal levels prior to and through the end of the race period. Data have shown that prior to the event, elevating levels of glycogen can improve performance in exercises that last longer than 1.5 h [19]. ‘Carb loading’ and consumption of meals with large quantities of carbohydrate (CHO) days and ~3 h prior to an event have been shown to improve performance [20]. However, on the day of the race, consumption of an elevated-CHO meal less than 60 min before the event can actually result in elevated blood glucose levels in some individuals, which can ultimately lead to hypoglycaemia following the body’s insulin surge. As a result, some data suggest that consuming too much CHO in the immediate pre-race time window can actually harm athlete performance. CHO intake and expenditure is a fine balance [21]. During the event, consumption of CHO improves exercise capacity [22]. The benefits of CHO ingestion during endurance exercise events extend beyond their simple metabolic value, surprisingly improving motor output and even showing value through CHO mouth rinses [23]. Such mouth rinses may also be beneficial during the race because of the simple fact that for some participants, the gastrointestinal discomfort from consuming liquids may be too great. The American College of Sports Medicine recommends that athletes consume 30–60 g of CHO during endurance events lasting longer than 1 h [24].

Another key component of nutrition is hydration. Dehydration can have devastating effects on the endurance athlete. However, as discussed earlier, a fine balance must be struck between loss of fluid and consumption of fluid, in order to avoid electrolyte abnormalities such as hyponatraemia [24]. Hydration before the race is important for all competitors, as both hyperhydration and euhydration states prior to the race have shown positive effects on athlete performance [24, 25]. Maintaining fluid balance during a race may, however, be challenging and should be guided by the expectation that an athlete should never lose greater than 2–3 % of bodyweight during a race lasting longer than 1.5 h. Hypertonic water with the addition of CHO and sodium improves the efficiency of water absorption within the body [26]. Such options are essential and should be provided at endurance events, contributing to athlete success and prevention of illness.

Nutrition is an area of strategic importance for endurance and sporting events, as it can impact the health and safety of participants. Special attention should be paid to individual athletes, as well as the nature of the event and the various portions of the course, involving, for example, biking, swimming and running [27]. Ultimately, nutritional intake of CHO, electrolytes and water are essential for an athlete to compete to their capacity.

## Cardiac Incidents

Mass endurance events bring together thousands of participants with individual medical histories, as well as risk factors for cardiac abnormalities known or unknown to the athlete. While several preventative measures and individual risks should be addressed by the athlete with his or her physician in advance, medical personnel need to be diligent, as cardiac events can occur in this setting. Recent advances in the response to cardiac arrest and improved outcomes point to endurance events CPR programs as examples of improved survival outcomes. In a study, published in the *New England Journal of Medicine*, of cardiac deaths in marathons and half-marathons occurring over 10 years, survival was reported in 30 % of runners experiencing cardiac arrest [28]. The main predictor in these positive outcomes was bystander-initiated cardiopulmonary resuscitation (CPR). In 2010, the American Heart Association introduced new guidelines emphasizing the need for high-quality chest compressions and ‘hands-only’ CPR [29]. The new guidelines have allowed for simplified training methods and proven greater likelihood of bystander response. Additionally, the utilization and placement of automated external defibrillators (AEDs) across areas of high risk during endurance events, with rapid deployment, are now considered part of the medical plan of these events [30]. The incidence of marathon- and half-marathon-related sudden cardiac death (SCD) is low, as it is seen in only 1 in 200,000 participants between these two distance events and is more common in men at a mean age of 42 years [28]. Clinical and autopsy evidence suggests that underlying structural pathology, such as hypertrophic cardiomyopathy, is associated with the deaths that occur, whereas ischaemic heart disease is the usual culprit for cardiac arrests in those who survive, although the overall incidence of both cardiac arrest and SCD is still low [31]. Additionally, the data show that the majority of these incidents occur during the last 4 miles of the event or at the finish line of running events [32]. In triathlons, the swim portion has presented similar incidents, which require identical measures in these circumstances.

In recent years, leading marathon and triathlon medical directors have implemented various programmes and protocols to ensure an even lower incidence of SCD. The World Marathon Majors (WMM) identified the use and distribution of AEDs as best practice at large-scale marathons [30]. Since 2010, several marathons have implemented various training programmes, which can be replicated globally to supplement any endurance medical plan. By providing video instruction training programmes on ‘hands-only’ CPR and AED use to all volunteers and runners, the likelihood of bystander response is increased. These programmes have successfully been implemented in several endurance events, such as the Boston, Houston and Chicago Marathons. Video training can be sent via emails to participants and volunteer staff as part of the informational tools that all endurance events utilize. Additionally, having educational opportunities such as videos or hands-on modules at the race ‘Expo’ and packet pick-up prior to race day are all examples of programmes currently in place. If local EMS providers are being utilized, AEDs can be distributed by various methods. EMS ambulances may have difficulties navigating mass endurance events; therefore, combining bike teams, golf-cart teams and EMS foot teams to supplement on-site ambulances may decrease response times [32]. Providing additional AEDs across the course at stationary aid stations where runners may present with cardiac complaints should also be considered. Finally, augmenting the pre-finish and finish-line area with teams and resources on hand to respond to these conditions may continue to show increased SCD survival at endurance events and lower death rates in comparison with the general community population.

## Command and Communications Structure

Organized endurance events—large or small—fall into the category of mass gathering events. It is recognized that the impact of the event may place a strain on the local community, its resources and public health, and, in some larger scaled events, may require state and federal support for public safety and security purposes [14]. According to the World Health Organization, a mass gathering is defined as “an organized or unplanned event where the number of people attending is sufficient to strain the planning and response resources of the community, state or nation hosting the event”. Additionally, the Federal Emergency Management Agency in the USA requires that any event with 1000 or more participants follow the National Incident Management Structure developed by the Department of Homeland Security to standardize incident management and response [33].

The planning and execution stages of mass gathering events such as endurance competitions involve various challenges. In all endurance events, medical incidents are expected, and in the sport of marathon, an injury rate of 2–10 % has been cited, based on environmental factors [3]. Being aware of the injury rates and types specific to an event is critical in the planning process. Most important, however, is implementation of a structure to manage the expected and unexpected incidents associated with mass events that can impact participants and public. In recent years, several examples in the running community have highlighted these concerns. The 2007 Chicago Marathon was cancelled by organizers mid-race because of extreme weather conditions, where temperatures escalated to 88 °F with high humidity [33]. The 2008 London Marathon involved a re-route around the 13-mile mark, due to a potential gas leak [34]. As a result of Hurricane Sandy, race officials for the 2012 ING New York City Marathon were forced to cancel their event because of public health concerns. Finally, the event at the 2013 Boston Marathon—a finish-line bombing—highlighted an even greater need for increased attention on planning for mass gathering events. Implementing measures and a structure can assist response to the expected associated incidents, as well as helping to mitigate the potential unexpected non-event incidents [32]. The endurance medical community has adapted and applied these concepts and models to the overall structure of mass event management. One example is the Chicago Model, which integrates an organizational structure, information systems and communication to enhance planning, preparedness and real-time response for mass gathering events [35].

## Organizational Structure

The use of an incident command structure (ICS) brings together a multitude of stakeholders integral to planning and execution of the event. The ICS is a top-down structure with a pre-designated chain of command, with one incident commander to maintain a line of authority [3]. This organizational structure enables coordinated planning and preparedness activities. Certain preparedness includes devising corresponding incident action plans for potential scenarios specific to the event. The ICS system facilitates planned execution of activities between all agencies (police and fire departments, event staff, security and public health officers) associated with the event [32]. The incident action plans should be reviewed and finalized in the planning phase prior to race day. As part of the ICS, it is integral to have a Unified Command (UC) centre on-site. Forward Command (FC) is a physical structure, which brings together the incident commanders of all involved agencies to share the same physical environment. This serves as the headquarters for communication and resource allocation. Being in the same physical environment enables more dynamic and direct communications, which accelerate the decision-making process [33, 34].

## Information Systems

During endurance events with mass participants and spectators spread across a wide area, obtaining accurate and real-time information can be difficult. Implementing innovative technology tools into an event will assist in clear and timely information flowing into the UC, which allows decision makers to manage the event. Throughout the event, individual agencies on the course should be responsible for collecting information from the course route and monitoring those data, using information systems in the FC. The organization within the FC should allow for collective information sharing in the physically shared command centre where dynamic decision-making occurs [35].

Several innovative tools exist and have been implemented in endurance events to assist in the collection of critical information, which allows greater communication in addition to decision processes. One tool is having a medical or participant tracking system. This system allows all stakeholders to monitor the health care services and respond to the needs in the field. For instance, the leaders in the FC can follow the state of all medical tents with this tracking system, which gives information regarding the number of runners being seen and their conditions. The system allows real-time awareness of injury rates and types, and thus surveillance for potential public health trends. The same system can be placed in off-course participant transport services, as well as in receiving medical facilities. This tracking system allows medical and event-organizing staff access to real-time data to provide accurate information to family or officials as needed. Meteorological data with newer technologies allow real-time information with regard to environmental factors that can impact the event. Having real-time access to these data in the FC allows greater understanding of the potential impact on participants and the general public, and allows for advance communication on changing conditions. For example, wet-bulb globe temperature (WBGT) devices placed across the course allow for information to be sent to the FC. Data provided by WBGT devices identify potential environmental strain and provide the ability to incorporate this information into any potential event decisions. Utilization of the city’s video surveillance system assists in monitoring large crowds, as well as verifying or identifying any incidents potentially impacting the event [33]. Global positioning system tracking on emergency medical vehicles and personnel has additionally been used to provide real-time accessible resources to incidents that require an immediate response, based on their location. Tracking of vehicles can also be used on transport vehicles for supplies, personnel and participants to improve awareness of locations and expected arrival times. The components of the information system coalesce in the FC, where organizers can identify acute events, communicate for timely coordination of responses and make time-dependent decisions to mitigate negative outcomes [32].

## Optimizing Communications

Having the ability to provide communications to participants, spectators and the public in real time prior to and during an event is critical for managing dynamic situations. Communication among all race personnel, EMS providers, local hospitals and participants is critical to prevent and respond to incidents during race day. The Chicago Model brings together the leaders of all involved agencies to achieve this level of communication successfully under these conditions. In cases of incidents—expected or unexpected—all agencies decide and act together, so the responses are generated quickly and in a coordinated fashion, avoiding duplication of responses [34].

**Fig. 2:**
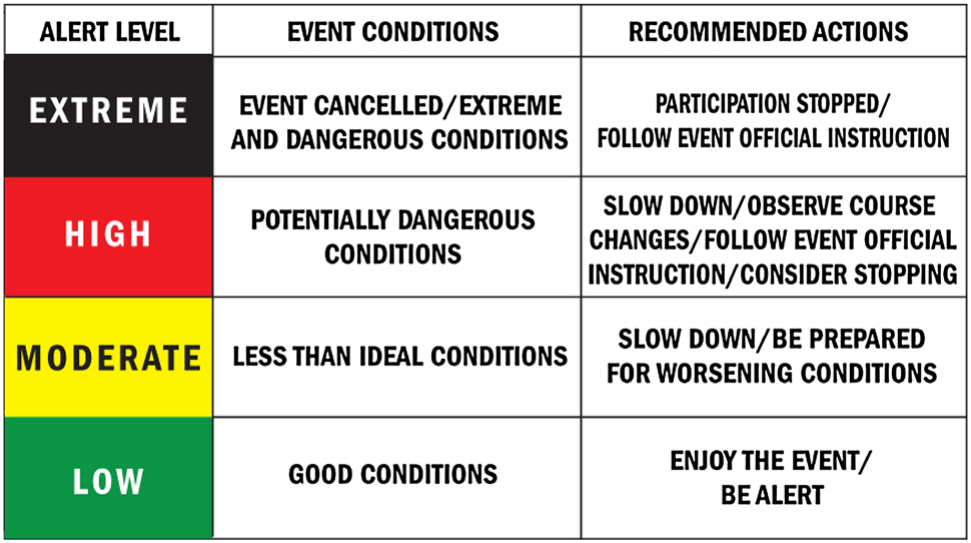
Event alert system (EAS). The EAS communicates the status of course conditions to event staff, participants and volunteers leading up to and on race day. Alert levels range from low (*green*) to moderate (*yellow*) to high (*red*) to extreme (*black*), based on a variety of factors, including weather conditions. All event staff, participants and volunteers are asked to familiarize themselves with the EAS colour indicators prior to the event and remain alert for directions from race officials, taking precautions to prepare for varying weather conditions. On race day, event staff, participants and volunteers are instructed to stay tuned to the EAS status via public address announcements and the colour-coded signs/flags at the start- and finish-line areas, and at each of the aid stations along the course

Designing an event alert system (EAS), which is a coloured flag tool, with advanced implementation as its purpose and use for an event, is a proven mechanism for communications before and on race day (Fig. 2). The EAS system was designed to improve communication with participants and all personnel involved with the event, regarding anticipated and changing weather conditions, including emergency situations [3]. Implementing layers of communication allows an unanticipated malfunction of one mode to be absorbed by the alternative methods without any noticeable disruption [32]. Information communication technologies (ICTs) have the potential to facilitate more widespread and potentially efficient communication systems to a large community in real time. This is often seen with the integration of mobile technology with voice communications, short messaging systems and even with social networking and Web 2.0 platforms [34]. Collecting and implementing emergency contact information on participants in advance, with the capability to message as needed via ICT, allows coordinated communication via the FC. Other mechanisms that are utilized add to the overall communication system. E-mail messaging, as well as use of social networking, provides additional platforms for participant updates and education. On race day, use of radios, as well as amateur radios, allows personnel to maintain lines of communication across the course, feeding into the FC. Variable message systems boards with dynamic messaging en route and controlled via the FC can disseminate potential changes on the course as they occur. If the event utilizes entertainment, such as DJs or bands, organizers can feed these groups pertinent emergency communications via the FC to be disseminated. Finally, utilization of the local police and fire personnel assigned to the event, and the city’s public address systems, are additional modes of streaming necessary information. The Chicago Model is successful because it is an iterative process. The coordinated information systems and incident command system facilitate a closed loop of communication between key decision makers in the ICS and responders and participants to effectively manage all scenarios [34].

## Conclusions

Endurance sports and competitions continue to draw athletes seeking newer and greater challenges. Utilizing innovative technologies while implementing medical advances in care for the unique conditions associated with these events is best practice. Ensuring that all medical providers understand potential collapse-associated injuries and rapid assessment and management of the more critical situations are vital for good outcomes. In addition, considerations for proper hydration and nutrition goals and practices may guide endurance outcomes, and thus both participant and medical staff education should be incorporated into the planning for such events. For a medical or race director, securing the necessary resources to support on-site medical staff in obtaining key medical information, in addition to providing care for participants, has proven benefits. Utilizing an incident command system, with all public and private stakeholders participating in advance planning, as well as on race day, allows for streamlined and rapid response to event-related or non-event-related incidents. Establishing and optimizing multiple communication systems, in addition to electronic medical participant tracking, allow for greater incident awareness, as well as rapid mass or specific dissemination of key information and thus public safety. Both event organizers and cities hosting mass endurance events should consider implementation of best-practice tools and medical protocols that are scalable and specific to their event.
